# Causes of Needlestick and Sharps Injuries When Using Devices with and without Safety Features

**DOI:** 10.3390/ijerph17238721

**Published:** 2020-11-24

**Authors:** Madeleine Dulon, Johanna Stranzinger, Dana Wendeler, Albert Nienhaus

**Affiliations:** 1 German Social Accident Insurance, Institution for the Health and Welfare Services (BGW), 22089 Hamburg, Germany; johanna.stranzinger@bgw-online.de (J.S.); dana.wendeler@bgw-online.de (D.W.); a.nienhaus@uke.de (A.N.); 2 Competence Center for Epidemiology and Health Services Research for Healthcare Professionals (CVCare), 20246 Hamburg, Germany

**Keywords:** needlestick injuries, safety-engineered devices, healthcare personnel

## Abstract

Safety-engineered devices (SEDs) have been developed to protect healthcare personnel (HCP) from needlestick and sharps injuries (NSIs). The aim of this study was to analyze NSIs associated with SEDs and non-SEDs among HCP in hospitals, medical offices and care facilities. Records from online questionnaires on NSIs were used. Causes of NSIs were compared for SED use and healthcare setting. A sample of 835 files was included. Injuries with SEDs accounted for 35.0% of all NSIs, whereas the proportions were higher in medical offices and lower in care facilities. NSIs in nurses were more often associated with SEDs than NSIs in physicians. NSIs from intravenous needles were associated with SEDs in more than 60% of cases in hospitals and medical offices and in about 30.0% of cases in care facilities. In contrast, suturing was associated with every fourth NSI in hospitals, of which fewer than 10.0% were associated with SEDs. In care facilities, SEDs were involved in 36.1% of NSIs during subcutaneous injections. NSIs during disposal accounted for 29.2% of total NSIs, of which 36.1% were associated with SEDs. Frequent reasons for SED-associated NSIs were technical problems, unexpected patient movement and problems during disposal. Our analysis shows that many NSIs are associated with SEDs. Continuous training is necessary in the handling and disposal of SEDs.

## 1. Introduction

In industrial countries, accidental needlestick and sharps injuries (NSIs) are the predominant sharps-related problem for healthcare personnel (HCP) [1]. In addition to the risk of becoming infected with a blood-borne virus and suffering chronic diseases, NSIs may also cause psychological effects, and the management of NSIs can be expensive [2]. It has been estimated that the annual incidence of NSIs is around 900,000 NSIs in five European countries (United Kingdom, France, Italy, Spain, Germany) [3]. The causes of NSIs are multifactorial and include elements such as types of devices and procedures, professional inexperience and lack of training and education in infection control and the principles of occupational health, improper management of sharps, poor organizational climate, high workload and fatigue [4,5]. In order to reduce the incidence of NSIs and to protect the safety of HCP, regulations on the prevention of sharps injuries in hospitals and the healthcare sector—such as the 2001 Needlestick Safety and Prevention Act and the 2010 EU Directive 2010/23/EU—have been issued to promote the dissemination of safety-engineered devices (SEDs) [6,7]. Safety features are designed to shield the needle or other sharp objects after use and are now available for blood collection systems, intravenous systems, hypodermic injection needles, suture needles, lancets and scalpels. Safety devices use different mechanisms (self-retractable needles, external shielding) or include a reuse prevention feature. Safety devices are divided into two main categories: passive and active. Passive devices do not require the manual operation of the device, as the safety element will activate during normal use. Active devices require activation of the safety function, such as activation of an integrated protection shield—such as a hinged cap—or by pressing an activation button to deploy the safety mechanism. Handling and activation are illustrated in Figures S1–S3.

After the introduction of SEDs, several studies have investigated the incidence of NSIs since 2001 and most of these have reported an overall reduction in NSIs due to the impact of SEDs [3,8,9,10,11]. Reviews have found low to moderate quality evidence of the inconsistent effects on NSIs during intravenous and phlebotomy procedures and hypodermic injections [12,13,14]. Nevertheless, there is no consistent evidence of a beneficial effect, which could be due to both confounding and bias [14].

Measures for preventing NSIs are governed in Germany by the Biological Agents Ordinance and are specified in detail in Technical Rule 250—Biological Agents in Health Care and Welfare Facilities (TRBA 250) [15]. The focus here is on the use of devices with SEDs, which became mandatory in Germany in 2013. Other protective measures are the avoidance of recapping, the use of sharps disposal containers and training in the handling of SEDs. Furthermore, it is recommended to implement ongoing surveillance of NSIs to identify the causes of NSIs and possible adverse effects. In Germany, HCP are required to record injuries in first-aid books or internal company documentation systems. There is no national record keeping system in Germany for NSIs with less than three days of inability to work. NSIs that lead to more than three days of inability to work have to be notified to the responsible agency for statutory accident insurance. Trend data from the German Social Accident Insurance in the Health and Welfare Services (BGW) show that in total a mean of 50,000 NSIs has been reported to the BGW each year over the last ten years [16]. The true number of NSIs sustained is still unclear, primarily due to underreporting [17].

The BGW is a statutory accident insurer, with the central task of preventing work-related accidents and occupational diseases and therefore supports its member companies by performing risk assessment after an NSI. Since April 2014, the BGW has provided a freely accessible online-NSI-tool on its website for NSI-related work accidents [18]. This online-NSI-tool was developed for two reasons: firstly, to support member companies in analyzing occupational accidents involving contact with blood and bodily fluids, and secondly, to enable the BGW to establish a computerized database of records on NSIs—in order to define new targets for preventive measures and to monitor the success or failure of these measures. In order to achieve these objectives, an online questionnaire was provided via a link to HCP using the online-NSI-tool on the BGW homepage. Information on the NSI was collected via standardized notification forms and stored anonymized on the BGW server, once the participant agreed to the conditions of the survey.

Although the body of literature on NSIs is large, little has been reported about the circumstances of injuries caused by SEDs. Therefore, the aim of the present study was to compare NSIs with and without SEDs. As NSI prevalence in non-hospital settings like long-term care facilities, home care or medical offices has only been investigated in a small number of studies, data were described separately for hospital and non-hospital settings.

## 2. Materials and Methods

The study was based on data collected via the online NSI tool on the BGW homepage between April 2014 and March 2019. A total of 2063 data sets were received and used as the data source.

**Figure 1:**
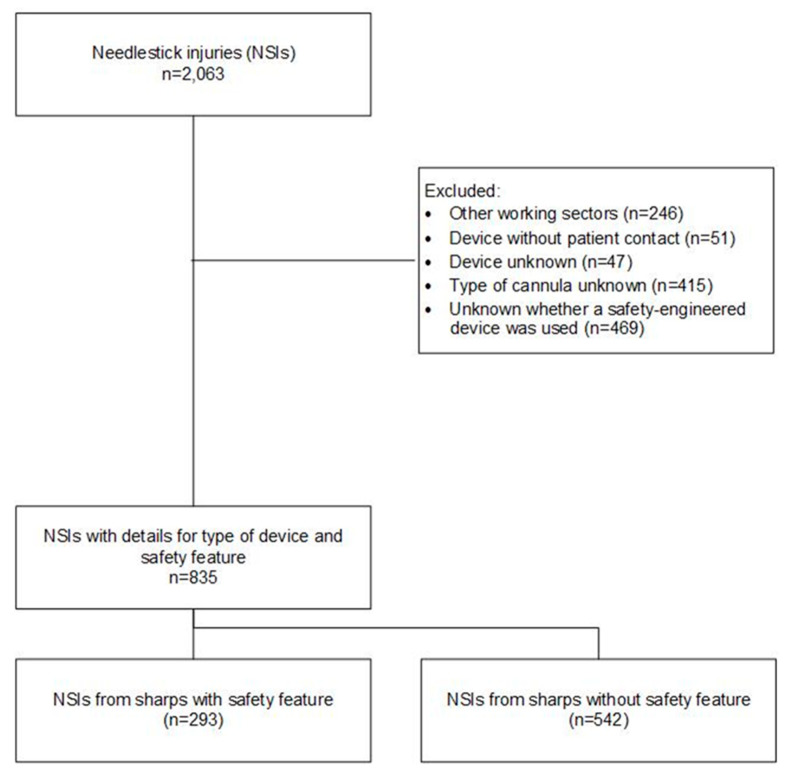
Flowchart for included and excluded cases.

An NSI was defined as an injury involving a contaminated sharp medical device, irrespective of whether or not the wound was bleeding. Exclusion criteria were: (1) participants not working in hospitals, medical offices or care facilities (11.9%); (2) devices that were not contaminated with blood (2.5%); (3) files without information on the device or without information on the type of cannula and whether this was with an SED or a non-SED (45.1%) (Figure 1).

Participants were characterized according to gender, age, profession, healthcare setting, and whether an SED was involved in the NSIs. In order to describe the circumstances of the accident, information was assessed on the type of device involved (“Which device led to your injury?”) and the type of activity when the injury occurred (“What were you doing when you suffered the injury?”). Information on the causes of accidents was collected (“What was the cause of your needlestick injury?”) and answers were grouped into the following categories: organizational failures (lack of sharps bin near the workplace, overfilled sharps bin, improper disposition of the device that caused the NSI; lack of training in handling SEDs); technical problems with the device (product failure, problems with the activation of the SED); unexpected movement of the patient; distraction from the surrounding area; lack of attention, and high workload (stress, time pressure, no opportunity for breaks). Frequencies were calculated separately for three healthcare settings: (1) hospitals, including clinics, rehabilitation clinics and dialysis centers; (2) medical offices, including any specialty, dentistry and laboratories; and (3) care facilities, including nursing homes for the elderly, care homes and outpatient care services. Data were analyzed descriptively using SPSS Version 25 (IBM Corp., Armonk, NY, USA). Multiple responses were analyzed by creating a multiple-response set. Data were presented as absolute frequencies for NSIs associated with SEDs and non-SEDs, separated by healthcare setting and as percentages of SED-associated NSIs within the total responses in each setting and calculated separately for each manifestation of the variables (characteristics, device, activity and reason).

## 3. Results

In total, 2063 records were received on the BGW server via the online NSI tool portal during the collection period, of which 835 (40.5%) satisfied the inclusion criteria. It is not possible to determine a reliable response rate, as the anonymized records received do not provide conclusive information about the subjects.

More than half the participants were working in hospitals (464 out of 835), almost 30.0% (246 out of 835) in medical offices and 15.0% (125 out of 835) in care facilities (Table 1). In 35.1% of NSIs (293 out of 835), an SED was used when the injury occurred. The corresponding percentages were 31.9% in hospitals, 45.1% in medical offices and 27.2% in care facilities. There were no evident differences between the employees injured by SED or non-SED, or with respect to either gender or age.

Physicians were involved in 28.7% of the total NSIs (240 out of 835), of which 27.5% were associated with SEDs (66 out of 240). Doctor’s assistants were almost equally as often injured by SEDs and non-SEDs. In hospitals, 35.0% of injuries sustained by nurses were associated with SEDs and in care facilities, 26.9% of injuries sustained by nursing staff (nurses and geriatric nurses) (25 out of 93) were SED-associated. Personnel in non-care job groups (cleaning, domestic services) accounted for only a few NSIs in all three settings, with varying proportions being associated with SEDs.

In hospitals and medical offices, needles for intravenous procedures were the devices most frequently involved in SED-associated NSIs, whereas in care facilities, this proportion was much lower (Table 2). Injuries from subcutaneous needles were associated with SEDs in 18.7% of NSIs in the total sample (23 out of 123). The respective proportion in care facilities was in line with this mean, however, needles for subcutaneous injections were involved in 56.0% of all NSIs in this setting (70 out of 125). NSIs from suture needles were caused by SEDs in only 6.2% in hospitals, whereas in medical offices and care facilities, the respective proportion was around 20%. Around 40% of NSIs from lancets were associated with SEDs in medical offices and care facilities and around 20% in hospitals. About 30% of NSIs from scalpels were associated with SEDs in medical offices, whereas in hospitals the respective proportion was only around 10%.

The most frequent procedures involved in NSIs in hospitals and medical offices were blood collection, suturing and disposal, whereas in care facilities, disposal was most involved, followed by administering subcutaneous injections. Blood collection was the most frequent activity for which NSIs were associated with SEDs (Table 3). Suturing and surgery were associated with 27.6% of all NSIs in hospitals (128 out of 464), but SEDs were involved in only a very few cases (7 out of 128). In medical offices, SEDs were involved in every third NSI during suturing. In care facilities, SEDs were involved in almost every second NSI during capillary blood collection and in every third NSI during subcutaneous injections. Injuries during disposal accounted for almost 30.0% of the total NSIs (244 out of 835). In those HCP using or handling an SED when injured, 30.5 % of the NSIs (89 out of 293) occurred during disposal. In those HCP handling a device without a safety feature, 28.6 % of the NSIs (155 out of 542) occurred during disposal. The respective proportions for SED-associated NSIs were higher in medical offices and lower in care facilities. Moreover, 28.6% of all NSIs during clearing up were associated with SEDs.

According to HCP in hospitals, NSIs were most frequently associated with a high workload (n = 154). On the other hand, HCP in medical offices reported that NSIs were equally as often associated with a high workload (n = 56) and organizational problems (n = 54) (Table 4). In both settings, SED-associated NSIs were associated with a high workload in around 30% of injuries. On the other hand, in medical offices, organizational problems were associated with almost every second NSI from SED. In hospitals and medical offices, unexpected patient movement was less often reported as the reason for NSIs but was nevertheless associated with more than 50% of SED-associated NSIs in both these settings. In care facilities, HCPs most often reported that unexpected patient movement was associated with NSIs (n = 35), although this was rarely the case for SED-associated NSIs. HCP in care facilities frequently reported that organizational problems were associated with NSIs (n = 32) but were only rarely regarded as a causal factor for SED-associated NSIs. The situation is quite different for technical problems. On the one hand, technical problems were reported to be the cause of the injury in less than 10.0% of NSIs overall. On the other hand, technical problems were involved in four of five SED-associated NSIs in hospitals and medical offices and in every second SED-associated NSI in care facilities. Lack of attention and distraction by the surroundings were not an important factor for NSIs in general or for SED-associated NSIs.

## 4. Discussion

The purpose of this study was to compare exposure data for injuries associated with SEDs and non-SEDs recorded by HCP working in hospital and non-hospital settings. There were originally more than 2000 records; about 40.0% of these fulfilled the inclusion criteria and included information on the cannula that triggered the accident and how the device was fitted with the safety feature. Thus, 835 NSIs were available for evaluation. In 35% of NSIs, an SED was being used when the accident occurred, whereas the proportions were higher in medical offices and lower in care facilities. A telephone survey of a comparable group of employees with NSIs found similar proportions of SED-related NSIs and reported that SEDs were present in around 80% of the workplaces in hospitals and medical offices and in 50% of the workplaces in care facilities [19]. However, this survey was performed in 2014 and thus only six months after SEDs had become mandatory in Germany. A hospital survey in Italy using routine data for the period from 2014 to 2016 found that 16% of NSIs were associated with SEDs [11].

In our study sample, NSIs in nurses were more often associated with SEDs than NSIs in physicians. Similar results have been reported by other studies, namely that NSIs associated with SEDs were more likely to occur in nurses whereas NSIs in physicians were more likely associated with non-SEDs [11,20]. With the data available to us, we were unable to conclude whether SEDs were less frequently used by physicians than by nurses.

It seems that in hospitals and medical offices, SEDs were apparently mostly used in blood sampling and other intravenous procedures, whereas non-SEDs were mostly used during suturing and surgery. Other studies have also shown that suturing with non-blunted sutures was the most common percutaneous injury associated with non-SEDs [20] Thus, non-safety suture needles were routinely used in 50% of hospitals in a Japanese study [9]. It is unclear why sutures without SEDs are still used in this way since a systematic review has concluded that there is high-quality evidence that the use of blunted suture needles appreciably reduces the risk for surgeons and their assistants [5].

The situation for NSIs from subcutaneous needles is quite different. Our data show that only one out of five NSIs from subcutaneous needles were associated with SEDs. This applies to all three settings, but this has a particular significance for care facilities as more than half of all NSIs in this setting were caused by subcutaneous needles. The reason for this is presumably that additional funding was not guaranteed for nadroparine and insulin needles equipped with a safety feature to be used in nursing homes for the elderly or in outpatient care. Only since November 2019 has it been guaranteed that the expenses for nadroparine and insulin needles with safety feature will be paid by health insurance funds for patients who need assistance with injections [21]. It is not yet clear whether this legal regulation will have an effect on the number of accidents with subcutaneous injection needles.

As the present study is cross-sectional, no conclusions can be drawn about trends in the use of SEDs. Two studies in the United States investigated the trends in NSIs among HCP over 15 years and the impact of SEDs after the Needlestick Safety and Prevention Act. These showed that the incidence for NSIs associated with SEDs was significantly reduced in the first five years after the Act, but changed less during the following years [20,22]. However, both studies demonstrated an increasing number of injuries with SEDs over time. A study performed in the Netherlands reported that the introduction of SEDs did not result in a decrease in the overall number of NSIs, but in a reduction in NSIs after the use of blood sugar needles with a passive system [23]. Other authors have also reported that the number of NSIs is reduced when passive SEDs are used [24]. Thus, the effect of passive SEDs has not yet been fully clarified [14]. For our study, there is no information on the security system used, as our experience from personal interviews has shown that participants were often very unsure as to whether they had used passive or active SEDs [19].

The present study found that approximately one-third of all NSIs were related to problems with disposal. However, it was shown by out data and by others that users of SEDs or non-SEDs were equally involved in disposal-related NSIs [11]. Other authors concluded that SED-related injuries were commonly linked to a combination of SEDs not being activated prior to disposal and the unsafe disposition of the device [23]. Other studies have shown that these practices did not only cause a risk for the person using the device, but also for co-workers such as cleaning personnel and other personnel responsible for clearing up [19]. It has been presumed that one reason for this unsafe handling of SEDs is that HCP perceived that the risk of NSIs was lower because of the safety feature, and that this resulted in the irresponsible disposal of needles [23].

Our data showed that HCP might face situations where they are not able to activate the safety mechanism properly due to circumstances such as unexpected patient movement during the puncture or in acute situations like an emergency. Other factors such as high workload, stress, inattention, fatigue and lapses of concentration, contributing to the risk of NSIs—as shown by our data and as mentioned by other studies—are common factors in the occurrence of NSIs [23,25,26].

With regard to training on the use of safety devices, it was shown by our previous study that training was only offered in two-thirds of workplaces equipped with SEDs and that 26% of HCP with SED-related NSIs mentioned a lack of training in the handling of SEDs as a cause for the injury [19]. The effectiveness of SEDs plus training in handling SEDs has been evaluated in different reviews. The authors found that a combination is more effective in preventing NSIs [27,28]. On the basis of our data, we can conclude that training should not only include handling SEDs but should also discuss problems in handling SEDs in unexpected situations and should emphasize the importance of correct disposal—independently of whether the devices have safety features or not. Our data did not include information about the organization of safety culture at the HCP’s workplace. However, it was shown that legislative regulations are an important component of NSI prevention, but the specific sharps regulations must be implemented on site and entail more than the introduction of SEDs and a one-off training session at the beginning [29].

Our study has some limitations. (1) All data on the accidents are based on self-reports by the participants. This information bias applies particularly to whether the relevant device involved in the NSI was an SED or a non-SED. In the case that a needle was misclassified as an SED, this would lead to overestimation of SED-associated NSIs. (2) Our data do not allow us to calculate incidence rates, as we do not know the number of SEDs used at the workplaces of the injured HCP. Therefore, it is not possible to estimate the preventive effect from the use of SEDs. Nevertheless, our study offers important insights, as it draws attention to potential measures to improve the use of SED. In addition, we showed that SEDs have been neglected in a large proportion of NSIs in Germany, even though the use of SEDs is mandatory.

## 5. Conclusions

The continued prevalence of NSIs is a multifaceted problem. With the implementation of needles with safety features, it was widely assumed that this measure would result in a decrease in the number of NSIs. Our data show that SEDs do not provide complete protection and are only effective if used and disposed of correctly. Injuries which occur despite the use of an SED may be due to the failure of activation. Therefore, it is necessary that hospital and non-hospital settings continue to offer training in handling SEDs. Regular training in safe working routines should be a matter of course for all involved professions and should include the safe disposal of devices, both with and without safety features. For all healthcare providers, the surveillance of exposure to NSIs must be improved. The analysis of the circumstances of NSIs can help to identify high-risk activities and help the authorities find the safest SEDs based on HCP experience and technological advances.

## Figures and Tables

**Table 1:** Characteristics of healthcare personnel with needlestick and sharps injuries (NSIs) by healthcare setting and device with or without a safety feature (SED) and SED-associated NSIs as a percentage of the characteristics total (n = 835).

	Hospital ^1^			Medical Office ^2^			Care Facility ^3^			% SED-Associated NSIs/Characteristics Total		
Characteristics	SED n = 148 n	Non-SED n = 316 n	Total n = 464 n	SED n = 111 n	Non-SED n = 135 n	Total n = 464 n	SED n = 34 n	Non-SED n = 91 n	Total n = 125 n	Hospital %	Medical Office %	Care Facility %
Gender												
Female	104	205	309	99	116	242	29	74	103	33.7	40.9	28.1
Male	41	106	147	11	16	27	5	17	22	27.9	40.7	29.4
Age (years)												
<21	9	24	33	18	15	33	3	5	8	27.3	54.5	37.5
21–30	53	107	160	26	40	66	9	27	36	33.1	39.4	25.0
31–40	25	82	107	16	26	42	7	21	28	23.4	38.1	25.0
41–50	27	50	77	26	27	53	9	17	26	35.1	49.1	34.6
>50	31	50	81	22	25	47	6	16	22	38.3	46.8	27.3
Profession												
Physician	50	141	191	16	33	49	0	0	0	26.2	32.7	0.0
Doctor’s assistant	10	12	22	61	74	135	0	1	1	45.5	45.2	0.0
Nurse	48	89	137	10	7	17	9	22	31	35.0	58.8	29.0
Geriatric nurse, auxiliary worker	3	3	6	1	0	1	16	46	62	50.0	100.0	25.8
Trainee nurse	30	30	60	16	18	34	7	7	14	33.3	47.0	33.3
Cleaning/kitchen personnel, other	7	12	19	7	3	10	2	8	10	36.8	70.0	20.0
% SED-associated NSIs related to setting	148/464 (31.9)			111/246 (45.1)			34/125 (27.2)					

Notes: SEDs, safety engineered devices. ^1^ Including hospitals, rehabilitation clinics and dialysis wards. ^2^ Including all specialist facilities, dentistry and laboratories. ^3^ Including nursing homes for the elderly, outpatient and hospice care, and others (e.g., facilities for the handicapped).

**Table 2:** Devices associated with needlestick and sharps injuries (NSIs) by healthcare setting ^1^ and device with or without a safety feature and SED-associated NSIs as a percentage of device types total (n = 835).

	Hospital			Medical Office			Care Facility			% SED-Associated NSIs/ Device Type Total		
Device Type	SED n = 148 n	Non-SED n = 316 n	Total n = 464 n	SED n = 111 n	Non-SED n = 135 n	Total n = 246 n	SED n = 34 n	Non-SED n = 91 n	Total n = 125 n	Hospital %	Medical Office %	Care Facility %
Intravenous needle ^2^	118	53	171	78	47	125	6	16	22	69.0	62.4	27.3
Subcutaneous (insulin) needle	7	39	46	0	7	7	16	54	70	15.2	0.0	22.9
Suture needle/surgical device	10	152	162	9	39	48	1	4	5	6.2	18.8	20.0
Scalpel	7	46	53	8	19	27	0	0	0	13.2	29.6	0.0
Lancet	2	7	9	9	14	23	11	13	24	22.2	39.1	45.8
Other device ^3^	4	19	23	7	9	16	0	4	4	17.4	43.8	0.0

Notes: SEDs, safety engineered devices. ^1^ Composition as described in Table 1. ^2^ Blood collection needle, butterfly needle, portacath needle. ^3^ Tweezers, intramuscular needle.

**Table 3:** Activities associated with needlestick and sharps injuries (NSIs) by healthcare setting ^1^ and device with and without a safety feature and SED-associated NSIs as a percentage of activities total (n = 835).

	Hospital			Medical Office			Care Facility			%SED-Associated NSIs/ Activities Total		
Activity When Injury Occurred ^2^	SED n	Non-SED n	Total n	SED n	Non-SED n	Total n	SED n	Non-SED n	Total n	Hospital %	Medical Office %	Care Facility %
Blood collection	62	17	79	41	21	62	0	2	2	78.5	66.1	0.0
Venous/arterial cannulation	22	36	58	8	4	12	0	6	6	37.9	66.7	0.0
Administering an injection (i.v., i.m., s.c.)	8	25	33	1	3	4	13	23	36	24.2	25.0	36.1
Capillary blood collection	4	4	8	2	9	11	8	9	17	50.0	18.2	47.1
Suturing/surgery	7	121	128	6	14	20	0	2	2	5.5	30.0	0.0
Disposal ^3^	35	64	99	42	46	88	12	45	57	35.4	47.7	21.1
Clearing up ^4^	16	36	52	13	29	42	3	15	18	38.8	31.0	16.7
Handling of sharps	4	13	17	6	5	11	0	2	2	28.5	54.5	0.0

Notes: SEDs, safety engineered devices. ^1^ As described in Table 1. ^2^ Multiple answers possible. ^3^ On the way to sharps disposal container, working on sharps disposal container, recapping and waste disposal. ^4^ Needle was left in inappropriate place (on trays, tables or beds).

**Table 4:** Reasons for needlestick and sharps injuries (NSIs) by healthcare setting and device with and without a safety feature ^1^ and SED-associated NSIs as a percentage of total reasons (n = 835).

	Hospital			Medical Office			Care Facility			% SED-Associated NSIs/ Reasons Total		
Reason ^2^	SED n	Non-SED n	Total n	SED n	Non-SED n	Total n	SED n	Non-SED n	Total n	Hospital %	Medical Office %	Care Facility %
High workload, stress	41	113	154	19	37	56	2	18	20	26.6	33.9	10.0
Organizational problem ^3^	33	60	90	26	33	54	6	28	32	36.7	48.1	18.8
Unexpected patient movement	48	42	90	28	19	47	8	27	35	53.3	59.6	22.9
Lack of attention	16	80	96	11	27	38	7	15	22	16.7	28.9	31.8
Technical problem, product failure	48	14	62	23	7	30	6	5	11	77.4	76.7	54.5
Distraction by surrounding	16	46	62	8	8	16	1	10	11	25.8	50.0	9.1

Notes: SEDs, safety engineered devices. ^1^ Composition as described in Table 1. ^2^ Multiple answers possible. ^3^ Lack of sharps bin at the usage location, overfilled sharps bin, improper disposition of the device; lack of training in handling SEDs.

## Supplementary Materials

The following are available online at https://www.mdpi.com/1660-4601/17/23/8721/s1, Figure S1: Device with integrated protection shield, active system, handling before cannulation (©BGW/Kröger + Gros); Figure S2: Device with integrated protection shield, active system, before activation of the safety function (©BGW/Werner Bartsch); Figure S3: Device with integrated protection shield, active system, after activation (©BGW/Werner Bartsch).
