# Supplementary material for: Causes of Needlestick and Sharps Injuries When Using Devices with and without Safety Features
Source: Int J Environ Res Public Health. 2020 Nov 24;17(23):8721. doi: 10.3390/ijerph17238721 (PMC7727709; doi:10.3390/ijerph17238721)
Supplement: Supplementary file 1 [file ijerph-17-08721-s001.pdf]

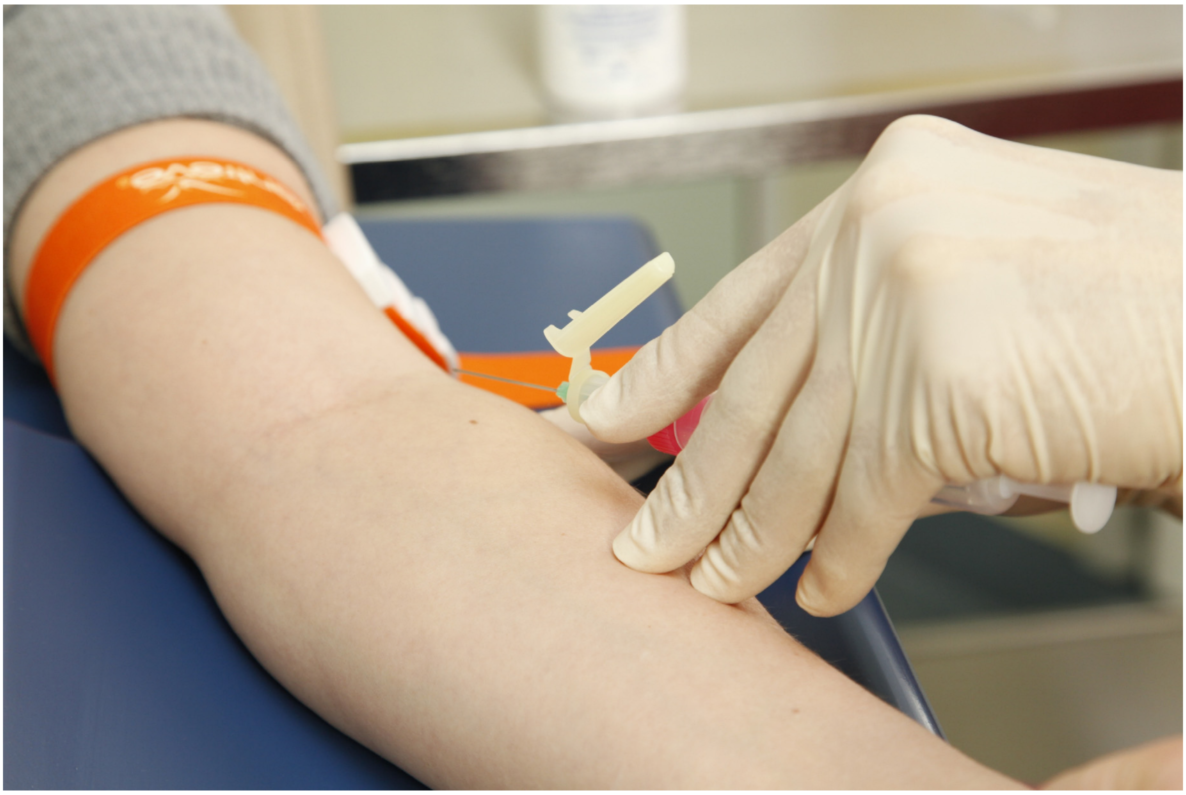

Figure S1. Device with integrated protection shield, active system, handling before cannulation (©BGW/Kröger + Gros)

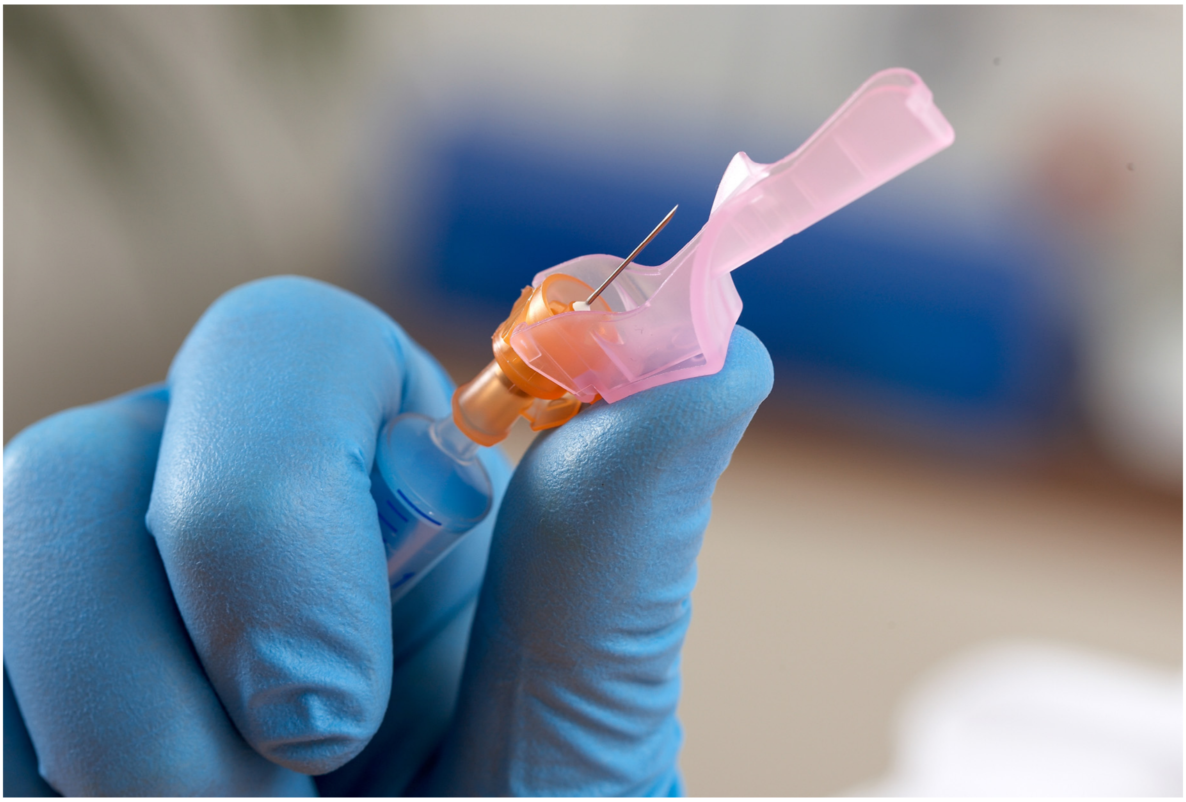

Figure S2. Device with integrated protection shield, active system, before activation of the safety function (©BGW/Werner Bartsch)

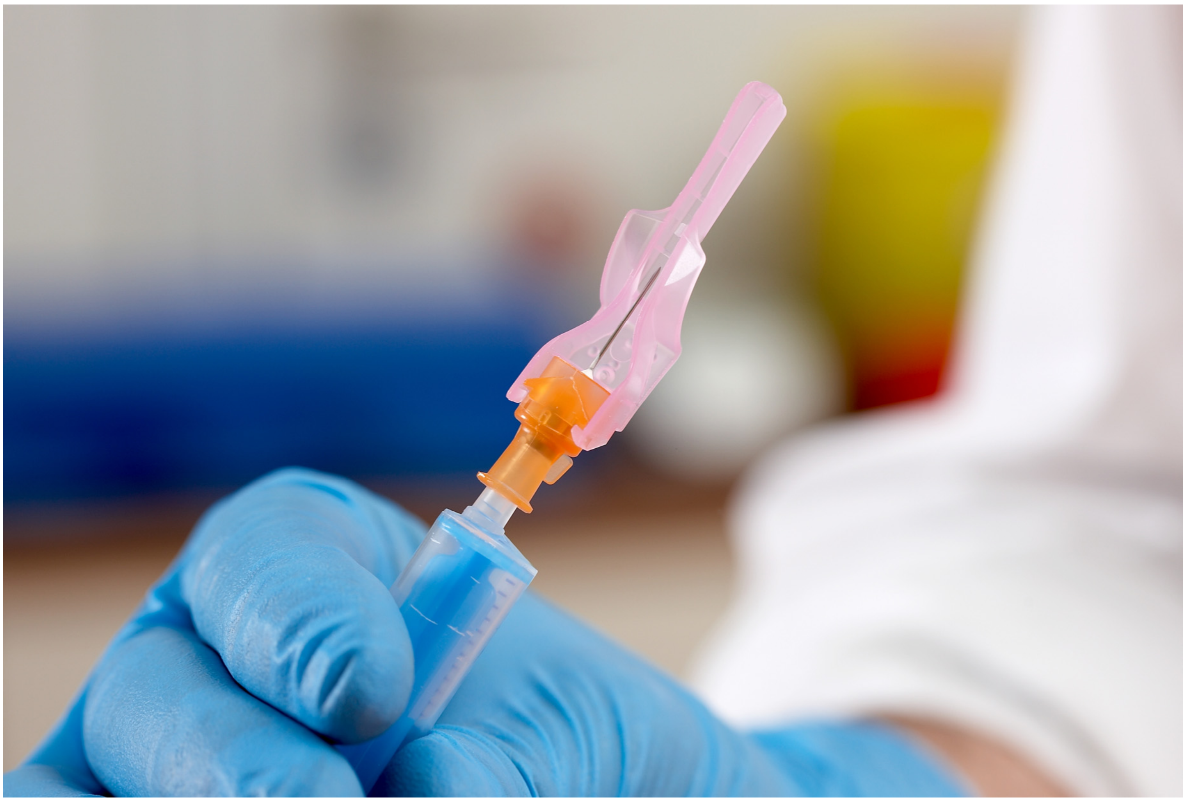

Figure S3. Device with integrated protection shield, active system, after activation (©BGW/Werner Bartsch)
